# Ocular Surface Disorders in Traffic Police Officers in the Union Territory of Puducherry, India: A Cross-Sectional Study

**DOI:** 10.7759/cureus.115533

**Published:** 2026-08-31

**Authors:** Priyadarshini Parthiban, Ezhilvathani T N, Banushree CS, Amudha . Purushothaman, Vijhaya Priya Thanasekaran, Renuka Sri Sai Peddireddi, Deepika Kalaimani

**Affiliations:** 1 Ophthalmology, Indira Gandhi Medical College and Research Institute, Puducherry, IND; 2 Pathology, Indira Gandhi Medical College and Research Institute, Puducherry, IND; 3 Ophthalmology, SRM Medical College Hospital and Research Centre, Chennai, IND

**Keywords:** conjunctival impression cytology, dry eye disease, nelson grading, ocular surface, traffic police

## Abstract

Background: Traffic police personnel are chronically exposed to vehicular emissions, environmental pollutants, and ultraviolet radiation, which may adversely affect their ocular health.

Aim: To evaluate the ocular surface disorders among traffic police personnel in Puducherry, India, using clinical examination and impression cytology.

Settings and design: A cross-sectional study was conducted among traffic police personnel (n=106) in the Ophthalmology Outpatient Department of the Indira Gandhi Medical College and Research Institute, Puducherry, India.

Materials and methods: A comprehensive ophthalmic examination, including the Ocular Surface Disease Index Questionnaire (OSDI), Schirmer’s test, tear film break-up time (TBUT), and tear meniscus height (TMH) test, was used to assess dry eye. Conjunctival impression cytology was performed to evaluate the microscopic epithelial alterations. Statistical analysis was performed using IBM SPSS Statistics for Windows, Version 21.0 (Released 2012; IBM Corp., Armonk, NY, United States). Associations were evaluated using the chi-square test, and the significance level was set at p < 0.05.

Results: According to Nelson's grading, 17% of the participants showed Grade 0, 47.2% showed Grade 1, 31.1% showed Grade 2, and 4.7% showed Grade 3. A statistically significant association was observed between the Nelson grade and the years of service in active field duty, the OSDI score, and TBUT (p < 0.05). Dry eye symptoms were present in 78.3%, and work-related stress was reported by 53.8%. The defined exposure in the study was chronic exposure to pollution, dust, and ultraviolet rays for a period of more than one year, and the outcome assessed was squamous metaplasia detected by conjunctival impression cytology, which shows a clear exposure-outcome relationship.

Conclusion: Chronic occupational exposure to air pollution and ultraviolet radiation contributes to dry eye disease and squamous metaplasia among traffic police personnel. Regular screening and preventive strategies are recommended.

## Introduction

Occupational health hazards are widely under-recognized in many countries, and exposures in the workplace are one of the major causes of morbidity and mortality [1]. Traffic police personnel are especially vulnerable because they are outdoors for extended periods of time, where they are exposed to vehicle emissions, particulate matter, dust, and ultraviolet radiation [2-4]. Pollutants such as particulate matter, carbon monoxide, nitrogen oxides, sulfur dioxide, and volatile organic compounds emitted by vehicle exhausts can cause oxidative stress, inflammation, and tear film instability in the eye [3]. Rapid urbanization and increasing traffic density in India have significantly increased pollution levels, severely impacting the health of outdoor workers. Almost 40% of traffic policemen have chronic cough and dermatitis, and more than 77% experience watering, redness, burning sensation, and decreased vision in their eyes [1,5]. A greater incidence of subclinical dry eye and ocular dryness has been strongly associated with urban air pollution in major Indian cities [6].

Although there is a high prevalence of symptomatic ocular surface disorders among traffic police personnel on active field duty in various cities in India, there is a significant lack of comprehensive clinical assessments of cytological damage [2,7]. To address the critical gap, this study not only relies on a self-reported symptom questionnaire but also includes an objective diagnostic method such as impression cytology of the conjunctiva to quantify the microscopic changes occurring in the conjunctiva [8].

There exists a critical need to quantify the extent of ocular morbidity caused by the chronic occupational exposure of traffic police. This study was structured with distinct primary and secondary objectives. The primary objective was to evaluate clinical ocular surface disorders, objective dry eye parameters, and conjunctival cytological changes, specifically early squamous metaplasia, among traffic police officers in Puducherry, India. The secondary objective was to explore the association between the duration of active field occupational exposure, years of service, and the ocular surface alterations. The prevalence of anterior and posterior segment ocular comorbidities, lifestyle behaviors, and work-related psychological stress within this vulnerable cohort was also documented.

## Materials and methods

Study design and ethical clearance

This is a cross-sectional study involving 106 traffic police personnel in Puducherry, India. The study was conducted in accordance with the Strengthening the Reporting of Observational Studies in Epidemiology (STROBE) guidelines. The study was approved by the Institute Ethics Committee of the Indira Gandhi Medical College and Research Institute (approval no. 548/IEC-38/PP-46/2023).

Study population and sampling

A universal sampling method was used in this study. A roster was compiled by the traffic police department based on their official daily attendance registers. From this register, eligible participants were sent to the Ophthalmology Outpatient Department at the Indira Gandhi Medical College and Research Institute. Two policemen were assessed each day. A total of 106 police officers were screened over a period of two months from January to February 2024. Informed consent was obtained from the participants, and their confidentiality was ensured. A detailed patient flow diagram illustrating the screening, exclusion, and final enrollment of the 106 participants is provided in Figure 1.

**Figure 1:**
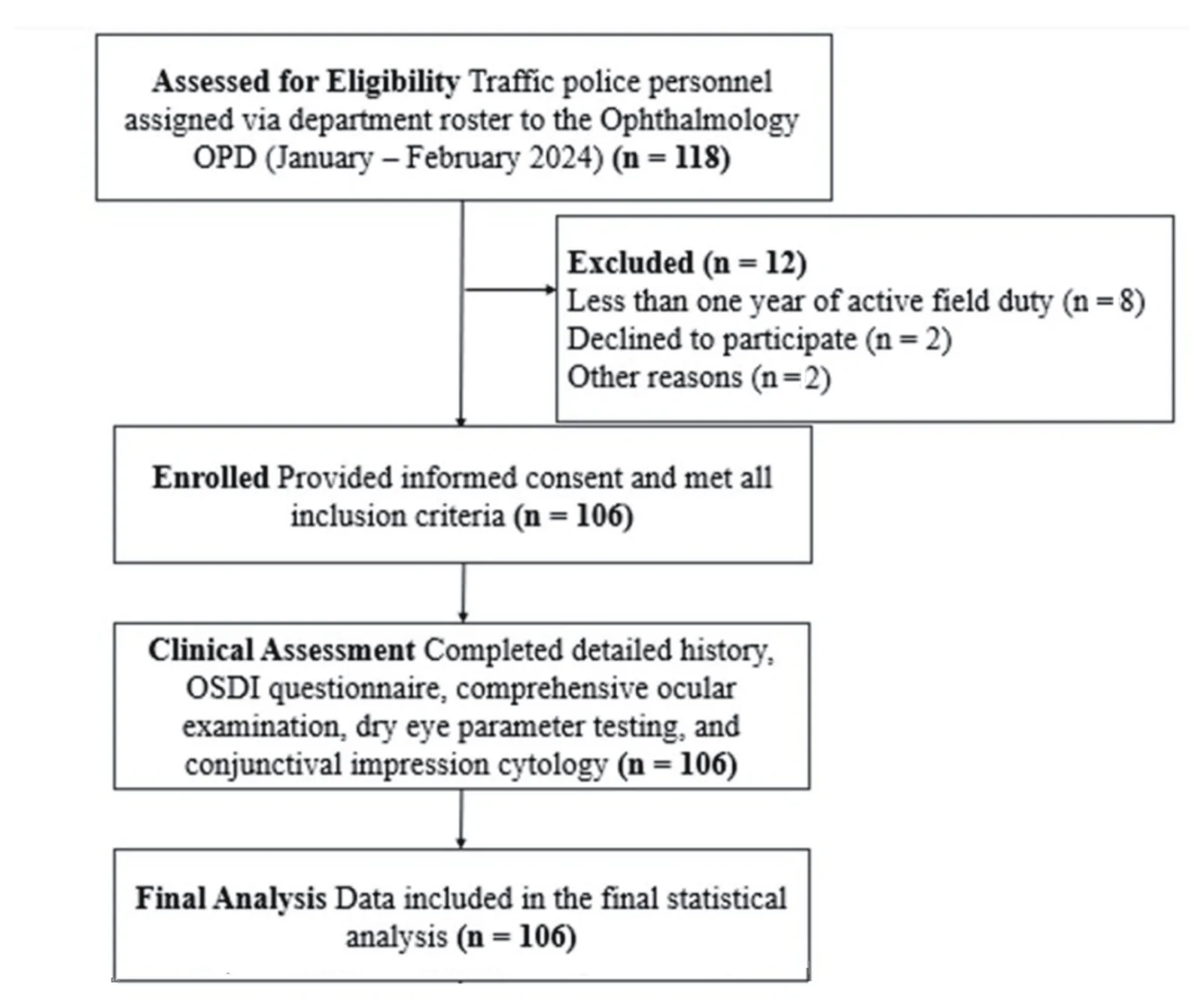
STROBE patient flow diagram. STROBE: Strengthening the Reporting of Observational Studies in Epidemiology; OPD: Outpatient Department.

Inclusion Criteria

Traffic police personnel of Puducherry who were actively performing field duty at the time of the study with a minimum duration of one year or more, were aged 20-60 years, were willing to participate in the study, and provided informed consent were included in the study.

Exclusion Criteria

Police officers with less than one year of active field duty, those who used contact lenses, those who had recently undergone any ocular surgery or refractive procedures, and those who had used topical ocular medications in the last three months were excluded from the study. Participants with known systemic diseases such as Sjogren's syndrome, rheumatoid arthritis, or sarcoidosis and those taking medications such as selective serotonin reuptake inhibitors (SSRIs) and tricyclic antidepressants were also excluded.

Data collection and clinical examination

A detailed history of all the participants was taken. Variables such as sex, age, and duration of service as traffic police were recorded. Participants were asked about the presence of ocular complaints or symptoms and their past medical and surgical history. The participants were also asked open-ended questions regarding work-related stress, sleep disturbance, and amount of water intake in a day. Their answers were recorded.

To establish a diagnosis of dry eye disease (DED) in accordance with the TFOS DEWS II guidelines, a positive screening symptom score (Ocular Surface Disease Index (OSDI) ≥ 13) was paired with at least one positive objective homeostasis marker [8]. The thresholds for homeostasis disruption were chosen as tear film instability (tear film break-up time (TBUT) < 10 seconds), tear volume deficiency (tear meniscus height (TMH) 0.2 mm or classified as "low"), or ocular surface compromise (Schirmer's test score ≤ 10 mm in 5 minutes). Meibomian gland dysfunction (MGD) was clinically diagnosed based on meibomian gland orifice plugging, lid margin vascularity, and compromised meibum expressability (Grade 1 or greater on digital expression of the central lower lid glands). To eliminate inter-observer bias and maintain strict methodological standardization, all clinical examinations, dry eye tests, and cytological slide preparations were performed by a single trained examiner for all 106 study participants. Allergic conjunctivitis was diagnosed clinically based on characteristic slit-lamp findings of conjunctival hyperemia, chemosis, and papillary reaction, corroborated by patient-reported itching, in keeping with standard clinical diagnostic criteria. All the participants were given the OSDI questionnaire [9], and their subjective symptoms were scaled according to the questionnaire as mild, moderate, or severe.

Routine ophthalmological examinations included visual acuity (VA) using a Snellen's VA chart, refraction, and slit-lamp evaluation of the anterior segment for ocular surface diseases such as blepharitis, allergic conjunctivitis, pterygium, and pinguecula. To objectively determine dry eye status without interference, TMH and TBUT were assessed first. These tests were followed by Schirmer’s test without topical anesthesia. The test was performed using standard 5 mm by 35 mm Whatman No. 41 filter paper strips. The paper strips were positioned in the lower conjunctival fornix for five minutes while the participants kept their eyes gently closed. Closed-eye testing is preferred in clinical research because it minimizes reflex tearing caused by strip movement, blinking, and ambient environmental exposure, thereby yielding highly reproducible measurements of basal tear secretion [10].

Conjunctival impression cytology and staining protocol

Conjunctival impression cytology specimens were obtained from the bulbar conjunctiva of both eyes using separate Millipore filter paper strips after the application of topical anesthesia. For each participant, two distinct smears were prepared from each eye: a wet-field smear and a dry-field smear. The wet-field smear was immediately fixed by immersion in 95% ethyl alcohol and subsequently stained using the Papanicolaou (PAP) technique to evaluate cellular features. The dry smear was air-dried and stained with Giemsa stain to highlight inflammatory cells and cellular morphology. The stained cytological smear was examined under a light microscope by the single evaluator and graded according to Nelson’s classification system (criteria outlined in Table 1) [11]. Participants finally underwent a dilated fundus examination and were treated for any identified ocular morbidities.

**Table 1:** Nelson’s grading system of conjunctival impression cytology.

Grade	Cell morphology	Goblet cells	Interpretation
0	Small, round epithelial cells	Numerous	Normal conjunctiva
1	Slightly enlarged cells	Mild decrease	Early squamous metaplasia
2	Larger polygonal cells	Marked decrease	Moderate squamous metaplasia
3	Large, flattened keratinized cells	Absent	Severe squamous metaplasia

Study outcomes

The primary outcomes of this study were objective conjunctival cytological changes (quantified via Nelson’s grading system) and clinical dry eye parameters comprising OSDI scores, MGD, TMH, and TBUT. The secondary outcomes of this study included other anterior segment pathologies such as pterygium, pinguecula, and allergic conjunctivitis; posterior segment findings, including diabetic retinopathy, hypertensive retinopathy, and glaucomatous disc suspects; and lifestyle stresses, including self-reported work stress, mean sleep duration, and daily water intake.

Statistical analysis

The data were presented as frequencies, percentages, means, and standard deviations. The continuous clinical variables (OSDI scores, Schirmer’s test, and TBUT) were categorized into discrete severity tiers prior to analysis. For all chi-square analyses, test statistics (χ² values) and degrees of freedom are reported alongside p-values. Effect size measures were quantified using Cramér's V to assess the strength of observed associations, with values interpreted according to Cohen's conventions (0.1 = small, 0.3 = moderate, 0.5 = large). Fisher's exact test was applied for comparisons where more than 20% of expected cell frequencies were less than five, specifically for the associations involving years of service, OSDI severity, and TBUT with Nelson's grading, and exact p-values are reported for these analyses. Statistical significance was set at p < 0.05. All analyses were performed using IBM SPSS version 21.0 (Released 2012; IBM Corp., Armonk, NY, United States).

## Results

Demographics

There were 106 traffic police officers, most of whom were male; 84 (79.2%) and 75 (70.8%) of participants were between the ages of 20 and 40 years. Most had one to five years of service (78, or 73.6%). Other demographic information and clinical characteristics are described in Table 2. Baseline VA test results are detailed in Table 3. All participants, including the single officer with a documented history of ischemic heart disease (Table 2), met the inclusion criterion of active field duty at the time of assessment; duty-type stratification (beat versus desk assignment) was not separately captured, as this fell outside the exposure classification used in this study. Officers with best-corrected VA below 6/6 (16/106 right eye; 10/106 left eye; Table 3) remained on active field duty; departmental visual-fitness and duty-reassignment protocols were outside the scope of data collection, and all participants with significant refractive or ocular findings were referred for further ophthalmological management as part of the study's clinical care protocol.

**Table 2:** Demographic details and clinical characteristics.

Variable	Category	Frequency (%)
Age (in years)	20-30	41 (38.70)
	31-40	34 (32.10)
	41-50	21 (19.80)
	>51	10 (9.40)
Gender	Female	22 (20.80)
	Male	84 (79.20)
Number of years of service as traffic police	1-5	78 (73.60)
	6-10	18 (17.00)
	11-15	3 (2.80)
	>16	7 (6.60)
Past history of diabetes mellitus	Absent	98 (92.50)
	Present	8 (7.50)
Past history of hypertension	Absent	97 (91.50)
	Present	9 (8.50)
Past history of ischemic heart disease	Absent	105 (99.10)
	Present	1 (0.90)
Past history of other diseases	Absent	103 (97.20)
	Acid peptic disease	1 (0.90)
	Bronchial asthma	2 (1.90)

**Table 3:** Baseline VA test characteristics. VA: visual acuity.

VA test		Frequency (n)
VA (right eye) unaided	6/6	67
	<6/6	39
VA (left eye) unaided	6/6	80
	<6/6	26
Best-corrected VA (right eye)	6/6	90
	<6/6	16
Best-corrected VA (left eye)	6/6	96
	<6/6	10

Conjunctival impression cytology showed goblet cells in 105 (99.1%) participants and squamous metaplasia in 75 (70.8%). Nelson grading revealed (criteria outlined in Table 1) Grade 0 in 18 (17%), Grade 1 in 50 (47.2%), Grade 2 in 33 (31.1%), and Grade 3 in 5 (4.7%) (Table 4).

**Table 4:** Conjunctival impression cytology findings.

Parameter	Category	Frequency (%)
Cellularity	Moderate	26 (24.50)
	High	80 (75.50)
Goblet cells	Present	
	Few	16 (15.10)
	Many	2 (1.90)
	Predominant	87 (82.10)
	Absent	1 (0.90)
Squamous cells	Present	
	Few	43 (40.60)
	Many	1 (0.90)
	Predominant	22 (20.80)
	Absent	40 (37.70)
Squamous metaplasia	Present	
	Few	44 (41.50)
	Many	4 (3.80)
	Predominant	27 (25.50)
	Absent	31 (29.20)
Anucleate squamous	Present	1 (0.90)
	Absent	105 (99.10)
Inflammatory infiltrate	Present	3 (2.80)
	Absent	103 (97.20)
Nelson grade	0	18 (17.00)
	1	50 (47.20)
	2	33 (31.10)
	3	5 (4.70)

Pterygium was present in 18 (17%), allergic conjunctivitis in 12 (11.3%), pinguecula in 11 (10.4%), and muddy pigmented conjunctiva in 26 (24.5%) of participants (Table 5).

**Table 5:** Anterior and posterior segment examination. MGD: meibomian gland dysfunction.

Eye component	Parameter	Frequency (%)
Lids	Canaliculitis	1 (0.9)
	Hordeolum internum	2 (1.9)
	Chalazion	1 (0.9)
	MGD	40 (37.7)
	Blepharitis	2 (1.9)
	Normal	60 (56.6)
Conjunctiva	Allergic conjunctivitis	12 (11.3)
	Bitot spots	1 (0.9)
	Episcleritis	4 (3.8)
	Nevus	2 (1.9)
	Muddy	26 (24.5)
	Pinguecula	11 (10.4)
	Pterygium	18 (17)
	Normal	32 (30.2)
Cornea	Nebular corneal opacity	1 (0.9)
	Normal	105 (99.1)
Lens	Normal	99 (93.4)
	Early cataract	7 (6.6)
Dilated fundus	Normal	86 (78.2)
	Glaucomatous disc suspect	12 (10.9)
	Mild non-proliferative diabetic retinopathy	4 (3.9)
	Moderate non-proliferative diabetic retinopathy	3 (2.7)
	Grade-1 hypertensive retinopathy	5 (4.5)

The dry eye prevalence was high, with 89 (84%) having an abnormal Schirmer's test, 90 (84.9%) having a decreased TBUT, and 83 (78.3%) experiencing OSDI symptoms (Table 6).

**Table 6:** Parameters to detect dry eye. TMH: tear meniscus height; TBUT: tear film break-up time; OSDI: Ocular Surface Disease Index; MGD: meibomian gland dysfunction.

Test	Category	Frequency (%)	Dry eyes (Yes), n (%)	Dry eyes (No), n (%)
Schirmer’s test	Severe (0-5 mm)	37 (34.9)	89 (84)	17 (16)
	Moderate (5-10 mm)	24 (22.6)		
	Mild (10-15 mm)	28 (26.5)		
	Normal (>15 mm)	17 (16)		
TMH	Low	74 (69.8)	91 (85.8)	15 (14.2)
	Medium	17 (16)		
	High	15 (14.2)		
TBUT	Low tear film stability (<5 s)	34 (32.1)	90 (84.9)	16 (15.1)
	Marginal tear film stability (5-10 s)	56 (52.8)		
	Normal tear film stability (>10 s)	16 (15.1)		
OSDI	Mild dry eyes (13-22)	31 (29.2)	83 (78.3)	23 (21.7)
	Moderate dry eyes (23-32)	35 (33)		
	Severe dry eyes (33-100)	17 (16)		
	Normal (0-12)	23 (21.7)		
MGD	No	66 (62.3)	-	-
	Yes	40 (37.7)		
Slit-lamp examination of blink rate/min (mean ± SD)		19.43±5.21	-	-

Common stressors on assessment were work-related stress (57, 53.8%) and sleep disturbance (35, 33%) (Table 7).

**Table 7:** Lifestyle factors affecting the participants.

Parameter	Frequency (%)
Work stress	
No	49 (46.2)
Yes	57 (53.8)
Sleep disturbance	
No	71 (67)
Yes	35 (33)
Number of hours of sleep, mean ± SD	6.35±0.96
Water intake per day in liters, mean ± SD	2.57±0.84

Years of service as a traffic police officer, OSDI questionnaire score for the symptoms, and dry eye parameters such as TBUT, Schirmer's test, TMH, and MGD were compared to Nelson's grading. A statistically significant association was present between years of service with Nelson grade (p = 0.014), OSDI-based symptom severity and Nelson grade (p = 0.002), and TBUT and Nelson grade (p = 0.012) (Table 8). Fisher’s exact test was applied to the cross-tabulations in which over 20% of expected cell frequencies were below five, including comparisons of Nelson grading with years of service (p = 0.014), OSDI severity (exact p = 0.002), and TBUT (exact p = 0.012) due to sparse cell counts in certain categories. Standard chi-square tests were utilized for the remaining parameters (MGD, TMH, and Schirmer's test), as their expected cell frequency assumptions were met. No significant correlation was observed between the Schirmer's test, TMH, and MGD with Nelson grading.

**Table 8:** Comparison of years of work, OSDI, and dry eye parameters with Nelson grade. Values in parentheses represent row percentages. p < 0.05 indicates statistically significant association. The non-significant associations (MGD, TMH, Schirmer's test) showed small-to-moderate effect sizes but lacked statistical precision to confirm these associations in the current sample. MGD: meibomian gland dysfunction; OSDI: Ocular Surface Disease Index; df: degrees of freedom; TMH: tear meniscus height; TBUT: tear film breakup time. ^*^Clinical tests that showed a statistically significant association.

Variable	Category	Nelson Grade 0	Nelson Grade 1	Nelson Grade 2	Nelson Grade 3	χ² value	df	p-value	Cramér's V
Years of work as traffic police	<5 years	18 (23.10%)	41 (52.60%)	16 (20.50%)	3 (3.80%)	15.68	9	0.014*	0.222
	6-10 years	0	6 (33.30%)	11 (61.10%)	1 (5.60%)				
	11-15 years	0	1 (33.30%)	2 (66.70%)	0				
	>16 years	0	2 (28.60%)	4 (57.10%)	1 (14.30%)				
OSDI severity	Mild	6 (19.40%)	10 (32.30%)	14 (45.20%)	1 (3.20%)	22.58	9	0.002*	0.266
	Moderate	4 (11.40%)	17 (48.60%)	10 (28.60%)	4 (11.40%)				
	Severe	1 (5.90%)	7 (41.20%)	9 (52.90%)	0				
	Normal	7 (30.40%)	16 (69.60%)	0	0				
OSDI dry eye	No	7 (30.40%)	16 (69.60%)	0	0	16.59	3	0.001*	0.396
	Yes	11 (13.30%)	34 (41.00%)	33 (39.80%)	5 (6.00%)				
MGD	No	12 (18.20%)	31 (47.00%)	18 (27.30%)	5 (7.60%)	4.02	3	0.260	0.195
	Yes	6 (15.00%)	19 (47.50%)	15 (37.50%)	0				
TBUT dry eye	No	5 (31.30%)	11 (68.80%)	0	0	10.92	3	0.012*	0.321
	Yes	13 (14.40%)	39 (43.30%)	33 (36.70%)	5 (5.60%)				
TMH dry eye	No	5 (33.30%)	9 (60.00%)	1 (6.70%)	0	7.55	3	0.056	0.267
	Yes	13 (14.30%)	41 (45.10%)	32 (35.20%)	5 (5.50%)				
Schirmer's dry eye	No	5 (29.40%)	10 (58.80%)	2 (11.80%)	0	5.80	3	0.121	0.234
	Yes	13 (14.60%)	40 (44.90%)	31 (34.80%)	5 (5.60%)				

Figure 2 shows a representative image of a conjunctival impression cytology smear in a few participants. Figure 3 shows some of the ocular surface disorders seen in traffic police officers in the present study.

**Figure 2:**
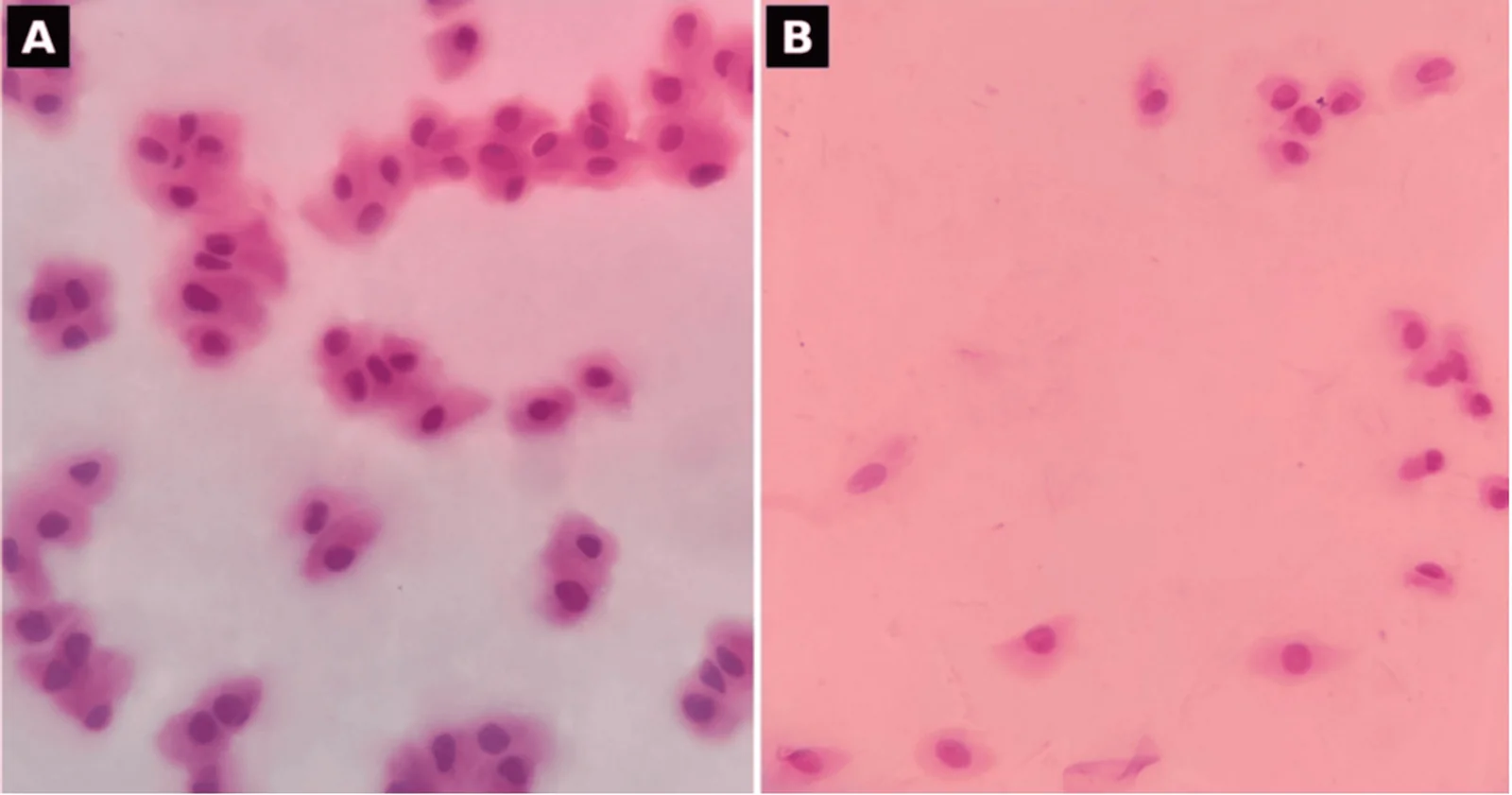
Conjunctival smear showing (A) extensive squamous metaplastic cells and (B) goblet cells in clusters and scattered individually.

**Figure 3:**
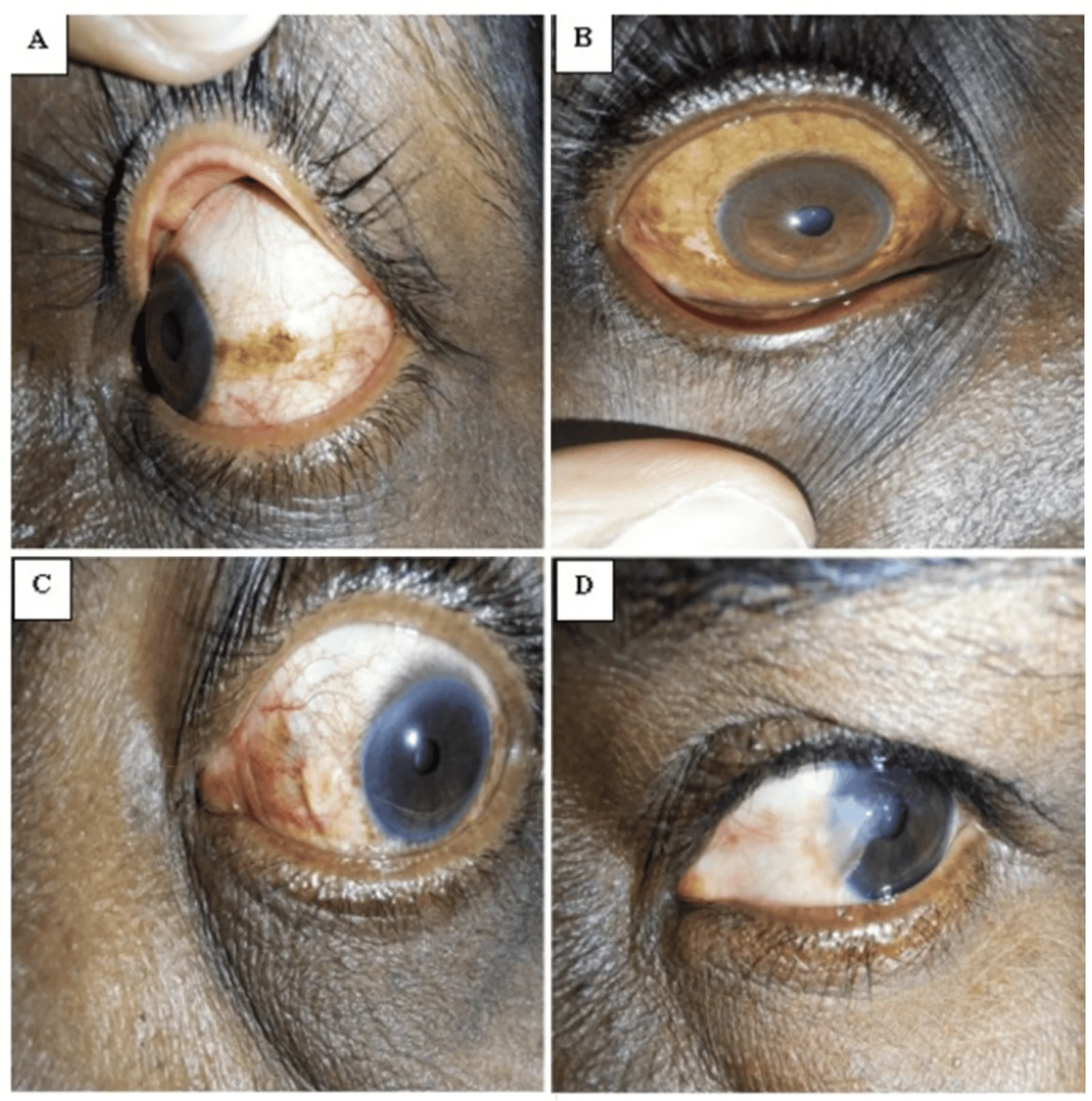
Some of the ocular surface disorders seen in traffic police officers. (A) Conjunctival nevus, (B) muddy pigmented conjunctiva, (C) pinguecula, (D) pterygium.

## Discussion

This study suggests a significant association between occupational exposure to traffic pollution and ocular surface epithelial damage. Traffic police officers with a longer service history demonstrated progressively higher grades of cytological alteration, mostly Nelson Grade 1 and Grade 2, indicating a potential exposure-accumulated correlation. It should be noted that years of service and age are likely correlated in this cohort; as age was not independently adjusted for in the bivariate analysis, this association may be partly confounded by age-related ocular surface changes (see Limitations). These findings are similar to the established data that link airborne pollutants to tear film instability and squamous metaplasia [12]. The increased incidence of ocular symptoms observed in India is similar to previous studies reporting significant eye irritation, subclinical dry eye, and decreased vision among traffic police personnel. In India, rapid urbanization heavily amplifies these occupational hazards [6,7]. This indicates that chronic exposure to urban pollution significantly reduces ocular health [13].

In conjunction with DED, exposure to urban pollution contributes to an increased frequency of other ocular surface disorders such as pinguecula, allergic conjunctivitis, and pterygium, likely caused by chronic irritation from ultraviolet rays and dust [7,8,14]. In addition, the detection of cataracts, retinal vascular pathologies, and glaucomatous optic neuropathy highlights the prevalence of systemic vascular disease in traffic police officers. Particulate pollution produces oxidative stress that increases vascular pathologies, indicating an urgent need for systemic and ocular health screening in traffic police personnel [3,4].

In studies conducted by Paudel et al. and Torricelli et al., it was found that more than 80% of all traffic police personnel exposed to pollution in their active field duty had symptoms of ocular surface disorders, of which a significant number of participants had severe symptoms [14,15]. A study by Torricelli et al. evaluated drivers and traffic controllers using OSDI scores and observed that the scores were mostly within the normal range [16].

Indian traffic police face a severe manpower shortage, inadequate daily water intake, long shifts, and absence of restroom facilities in intersections [17]. Dehydration and sleep disturbance can also significantly reduce the tear secretion and exacerbate dry eye symptoms [6,12]. The strength of this study is the holistic methodology, which combines both a subjective questionnaire-based study and objective tests to detect dry eyes, such as the Schirmer's test, TBUT, and TMH, along with conjunctival impression cytology, which is considered the gold standard for the evaluation of early squamous metaplasia [8]. In addition to this, a detailed ophthalmic examination using a slit lamp was done to look for various ocular surface disorders such as pterygium, pinguecula, etc., along with a fundus examination to identify other ocular pathologies such as cataract, diabetic retinopathy, hypertensive retinopathy, glaucoma, etc.

Implementation of periodic comprehensive ocular health screening, mandatory ultraviolet protective eyewear, and improvement of occupational infrastructure, particularly by providing sunshades, outposts, and accessible restroom facilities to the traffic police personnel, is necessary by the government authorities. This recommendation is supported by evidence linking systemic hydration status to tear film stability and dry eye risk, providing a plausible mechanistic link between restroom/hydration access and ocular surface disorder in this cohort [18].

Limitations

This study is cross-sectional, which restricts the ability to establish a temporal causality between the environmental exposure and ocular surface damage. The lack of an unexposed control group (such as indoor administrative workers) limits our ability to definitively isolate the primary etiology. The sample size (n=106) precluded multivariate regression to control for critical confounding factors, including age, sex, smoking, and use of protective eyewear. The male-dominated cohort restricts the generalizability of these findings to female traffic officers. Additionally, as a single-center study conducted within one union territory, the geographic and climatic generalizability of these findings to traffic police personnel in other regions of India, or with differing pollution and climate profiles, remains to be established. The Nelson Grade 3 (severe) subgroup contained a small number of participants (n=5), which limited the statistical power and reliability of comparisons involving this category; larger, ideally multicentric, studies with greater sample sizes are recommended to confirm these associations and to permit finer trend analysis. An age- and sex-matched case-control design comparing traffic police officers with an unexposed indoor cohort would more robustly establish the association between duration of occupational exposure and ocular surface damage than the present cross-sectional design. Lifestyle variables, including work-related stress, sleep duration, and daily water intake, were captured through self-report rather than validated instruments or objective measurement, introducing potential recall and reporting bias. VA and best-corrected VA were recorded (Table 3), but spectacle/refractive-correction status was not further stratified in the dry eye analysis, and its potential influence on ocular surface symptoms could not be independently assessed. Similarly, while posterior segment findings such as diabetic and hypertensive retinopathy are primarily systemic microvascular manifestations, this study did not formally analyze their relationship with ocular surface parameters; this remains an area for future targeted investigation.

## Conclusions

Traffic police personnel appear to be highly susceptible to ocular surface disorders because of their long-term exposure to ultraviolet rays and environmental pollutants. The prolonged exposure to the environmental pollution creates a dose-response relationship. The findings of this study suggest a strong correlation between the duration of active field duty and the severity of conjunctival epithelial damage. These personnel suffer from a high prevalence of DED and related comorbidities such as pterygium, allergic conjunctivitis, and pinguecula. To reduce the extent of occupational comorbidities in traffic police personnel's health strategies, infrastructural interventions are highly necessary. Increased awareness of risk factors and regular comprehensive eye examinations are necessary for early disease identification and treatment. Modifying stressors by providing accessible restrooms at traffic intersections is critical to promote adequate hydration and to alleviate the dry eye symptoms.
